# Essential Oils from Cameroonian Aromatic Plants as Effective Insecticides against Mosquitoes, Houseflies, and Moths

**DOI:** 10.3390/plants11182353

**Published:** 2022-09-09

**Authors:** Joice G. Nkuimi Wandjou, Cecilia Baldassarri, Marta Ferrati, Filippo Maggi, Roman Pavela, Nole Tsabang, Riccardo Petrelli, Renato Ricciardi, Nicolas Desneux, Giovanni Benelli

**Affiliations:** 1 Chemistry Interdisciplinary Project (ChIP), School of Pharmacy, University of Camerino, 62032 Camerino, Italy; 2 Crop Research Institute, Drnovska 507, 161 06 Prague 6, Czech Republic; 3 Department of Plant Protection, Czech University of Life Sciences Prague, Kamycka 129, 165 00 Prague 6, Suchdol, Czech Republic; 4 Department of Animal Biology, University of Dschang, Dschang, Cameroon; 5 Department of Agriculture, Food and Environment, University of Pisa, Via del Borghetto 80, 56124 Pisa, Italy; 6 Université Côte d’Azur, INRAE, CNRS, UMR ISA, 06000 Nice, France

**Keywords:** *Monodora myristica*, *Aframomum citratum*, *Xylopia aethiopica*, *Culex quinquefasciatus*, *Musca domestica*, *Spodoptera littoralis*, Culicidae, Muscidae, Noctuidae, geraniol, sabinene, α-pinene, *p*-cymene, α-phellandrene, β-pinene

## Abstract

Recently, spices have attracted the attention of scientists and agrochemical companies for their potential as insecticidal and acaricidal agents, and even as repellents to replace synthetic compounds that are labeled with detrimental impacts on environment and human and animal health. In this framework, the aim of this study was to evaluate the insecticidal potential of the essential oils (EOs) obtained from three Cameroonian aromatic plants, namely *Monodora myristica* (Gaertn.) Dunal, *Xylopia aethiopica* (Dunal) A. Rich., and *Aframomum citratum* (J. Pereira) K. Schum. They were produced by hydrodistillation, with yields of 3.84, 4.89, and 0.85%, respectively. The chemical composition was evaluated by GC-MS analysis. The EOs and their major constituents (i.e., geraniol, sabinene, α-pinene, *p*-cymene, α-phellandrene, and β-pinene) were tested against the polyphagous moth pest, i.e., *Spodoptera littoralis* (Boisd.), the common housefly, *Musca domestica* L., and the filariasis and arbovirus mosquito vector, *Culex quinquefasciatus* Say. Our results showed that *M. myristica* and *X. aethiopica* EOs were the most effective against *M. domestica* adults, being effective on both males (22.1 µg adult^−1^) and females (LD_50_: 29.1 µg adult^−1^). The *M. myristica* EO and geraniol showed the highest toxicity on *S. littoralis,* with LD_50(90)_ values of 29.3 (123.5) and 25.3 (83.2) µg larva^−1^, respectively. Last, the EOs from *M. myristica* and *X. aethiopica*, as well as the major constituents *p*-cymene and α-phellandrene, were the most toxic against *C. quinquefasciatus* larvae. The selected EOs may potentially lead to the production of cheap and effective botanical insecticides for African smallholders, although the development of effective formulations, a safety evaluation, and an in-depth study of their efficacy on different insect species are needed.

## 1. Introduction

Spices are aromatic plants or their specific parts, including bark, flowers, fruits, and seeds, owning a peculiar aroma given by flavoring-odorous compounds such as volatile terpenes and phenylpropanoids. Aromatic plants have attracted the attention of explorers since ancient civilizations because of their numerous culinary and medical applications [1]. Most of the plants classified as ‘spices’ come from aromatic plants growing in tropical regions, notably Asia and Africa, from where they have been brought to Europe since ancient times, becoming an important economic activity; the main examples are cinnamon (*Cinnamomum verum* J. Presl) and clove (*Syzygium aromaticum* (L.) Merr. & L.M. Perry), which are still sold today and used all over the world for numerous purposes. 

Nowadays, aromatic plants have numerous applications in the food industry, perfumery, and pharmaceuticals. In addition, some attracted the attention of scientists and agrochemical companies for their potential as insecticidal and acaricidal agents and even as repellents [2,3,4,5]. Aromatic plants contain essential oils (EOs) that have shown a wide insecticidal spectrum with limited impact on non-target organisms such as pollinators and biological control agents [6,7,8,9,10]. They can act as fumigants, contact toxicants, antifeedant and repellent agents [1,11,12,13,14,15,16], and could be an effective alternative to regular chemical pesticides [17,18]. Thus, developing commercial insecticides using spice derivatives could be a good strategy to replace synthetic compounds, which are nowadays labeled with detrimental impacts on the environment and human and animal health [19].

In this framework, we reported a comprehensive evaluation of the insecticidal potential of the essential oils obtained from three Cameroonian aromatic plants, namely *Monodora myristica* (Gaertn.) Dunal, *Xylopia aethiopica* (Dunal) A.Rich., and *Aframomum citratum* (J. Pereira) K. Schum., against a panel of economically relevant insect vectors and agricultural pests.

*M. myristica*, also called calabash nutmeg or African nutmeg, is a perennial plant from the Annonaceae family. This plant is widespread in Africa, Asia, Central and South America, and Australia, and is unable to grow out of its natural habitat [20]. In Africa, it is commonly found in Cameroon, Nigeria, Ghana, Liberia, Angola, Uganda, and west Kenya where its seeds, because of their aromatic flavor, are used as a spice in food preparation [20,21]. These seeds of about 1.5 cm long have demonstrated many healthy properties such as being antihypertensive, anti-headache, stomachic, and antiemetic [20,21].

*X. aethiopica*, known as Africa guinea pepper or Ethiopian pepper, is also a plant from the Annonaceae family, native to tropical Africa [22]. This plant is restricted mainly to the Guinean zone, from Senegal to Angola and Mozambique in the east; it grows in wet and swampy soils [23]. In Cameroon, it is found in the forest, forest edges, forest galleries, and savannah scrubs. The fruits of this plant have good nutritional value. They have demonstrated beneficial health effects and are used in the treatment of cough, bronchitis, and dysentery [22,24]. The extracts of this plant have also shown antibacterial, antifungal, and antiplasmodial effects [25].

*A. citratum*, belonging to the Zingiberaceae family, is a perennial herb with an underground rhizome [26]. This plant originates from Central and West Africa, where it is used as an aphrodisiac and in the treatment of bacterial infections, malaria, and cancers [26]. Its fruit is an ovoid capsule of 3 cm in diameter extended by the long persistent tube of the calyx, with many seeds of about 3 mm in diameter, harmonious, and contained in a white pulp. The seeds are also used as a food seasoning in Cameroon. 

Despite the ethnopharmacological interest in these three plant species, research efforts to shed light on the potential insecticidal properties of their EOs are limited, and mainly focused on the use of *X. ethiopica* EO against stored product insect pests [27,28,29,30,31,32]. In this framework, herein we evaluated the EOs extracted from *M. myristica*, *X. aethiopica*, and *A. citratum* against a highly polyphagous moth pest, i.e., the cotton leaf worm, *Spodoptera littoralis* (Boisd.) [33], and two insects of medical and veterinary importance, i.e., the common housefly, *Musca domestica* L., and the filariasis and arbovirus mosquito vector, *Culex quinquefasciatus* Say, for which effective and sustainable control tools are still needed [34,35]. The EOs were analyzed through GC-MS analysis, and the toxicity of the main constituents of each EO was evaluated on the three insect targets to evaluate the role of major compounds in determining the insecticidal activity of the whole EOs.

## 2. Results

### 2.1. Essential Oil Yield and Chemical Composition

The hydrodistillation of the seeds has given yields of 3.84% for *M. myristica*, 4.89% for *X. aethiopica*, and 0.85% for *A. citratum*. The chemical compositions of the three EOs are reported in Table 1. We identified 35 compounds for *M. myristica*, 55 compounds for *X. aethiopica*, and 23 compounds for *A. citratum*, representing 92.9, 99.6, and 99.9% of the total composition, respectively. The main volatile compounds present in *M. myristica* were *p*-cymene (32.8%), α-phellandrene (32.3%), α-pinene (7.6%), limonene (4.4%), and myrcene (4.3%), while in the *X. aethiopica* EO we found sabinene (26.1%), β-pinene (17.4%), germacrene D (9.7%), α-pinene (9.6%), β-phellandrene (6.2%), and terpinene-4-ol (6.1%). The major compound in the *A. citratum* EO was geraniol (85.6%) followed by a minor percentage of β-pinene (5.4%).

### 2.2. Insecticidal Activity

The effects of *M. myristica*, *X. aethiopica*, and *A. citratum* EOs and their major compounds on *M. domestica* adult mortality are presented in Table 2. All our tested EOs were toxic to the insect species included in this study. However, significant differences were observed between the EOs. EOs from *M. myristica* and *X. aethiopica* showed the highest efficacy, with an LD_50(90)_ of 29.1(137.6) and 30.7(164.2) µg adult^−1^ on females, respectively, and 22.1(127.6) and 31.5(178.5) µg adult^−1^, respectively, on males (Table 2).

The efficacy of *M. myristica* EO against houseflies was roughly equivalent to that of the major compound *p*-cymene, with the LD_50_ estimated as 28.4 µg adult^−1^ for females and 32.6 µg adult^−1^ for males. The other major compounds showed better efficacy compared to EOs, with special reference to the LD_50_ values estimated on males, which were more sensitive; nevertheless, LD_90_ values were approximately on the same level as those estimated for the EOs.

The tested EOs showed promising efficacy also against *S. littoralis* larvae (Table 3). Again, the highest efficacy was provided by the *M. myristica* EO with LD_50(90)_ estimated as 29.3(123.5) µg larva^−1^. However, given the overlapping confidence intervals (CI_95_), it is impossible to determine with certainty whether the efficacy was significantly better. Among individual major compounds, a significantly better efficacy was shown by geraniol, which achieved a significantly lower LD_50_ (=25.3 µg larva^−1^), as well as LD_90_ (=83.2 µg larva^−1^), if compared to other tested substances (Table 3). 

Last, the efficacy of the three EOs against *C. quinquefasciatus* larvae was very promising (Table 4). Higher efficacy was observed for *M. myristica* and *X. aethiopica* EOs, with LC_50(90)_ estimated as 35.3(66.1) and 47.0(78.4) µg mL^−1^, respectively. Concerning tests carried out with major constituents, the highest efficacy was observed for *p*-cymene and α-phellandrene, with LC_50_ estimated as 26.8 and 36.8 µg mL^−1^, respectively (Table 4).

## 3. Discussion

### 3.1. Essential Oil Chemical Composition

The three EOs were analyzed using GC–MS, and the relative content of each component was determined by comparing the ratio of the peak area of each detected compound with the total area of all detected compounds. It resulted in the identification and quantification of both the major and minor compounds that are responsible for the insecticidal activity, acting synergistically. Regarding *M. myristica* harvested in the locality of Dschang in west Cameroon, Massodi et al. [21] found that the most abundant compounds in the EO were α-phellandrene (52.2%), followed by α-pinene (6.3%), myrcene (4.4%), limonene (3.7%), and α-thujene (2.9%). Similarly, the most represented class reported in this latter study was that of monoterpene hydrocarbons, representing 69.5% of the total composition. Meffo Dongmo et al. [26] identified 20 compounds in *M. myristica* EO from Bafoussam locality (west Cameroon) with the predominant compound as in the case of Massodi et al. [21] being α-phellandrene (61.5%) followed by germacradienol (7.9%) and δ-cadinene (4.2%). The analysis of this EO from the forest of Lobaye in Central African Republic by Koudou et al. [36] showed that α-phellandrene (34.4%) and *p*-cymene (22.2%) were the most abundant among 30 identified compounds. While Owokotomo and Ekundayo [37] have demonstrated that the EO of *M. myristica* seed harvested in Iwaro-Oka, Nigeria, was rich in germacrene-D-4-ol (25.48%), tricyclo [5.2.1(1,5)] dec-2-ene (13.35%), δ-cadinene (11.09%), and linalool (15.10%) on 22 identified compounds.

The chemical composition of *X. aethiopica* from Cameroon (Kribi-Southwest) and Chad (Gobe) reported by Bakarnga-Via et al. [38] demonstrated that this EO was mainly constituted by monoterpene hydrocarbons, i.e. 72.4 and 64.8%, respectively, a percentage close to that we found in our study (69.8%); nevertheless, the EOs qualitative profile was different. In the latter study, the main compounds were β-pinene (24.6.9–28.2%), terpinen-4-ol (10.0–15.1%), sabinene (4.8–14.5%), β-phellandrene (5.8–10.4%), and γ-terpinene (4.9–5.7%) [38]. Another study of this oil from the Ivory Coast revealed that the most represented compounds were β-pinene (20.5%), α-pinene (17.8 %), 1.8-cineole (7.4%), and α-phellandrene (5.6%) [39]. Keita et al. [23] reported that the dried fruit powder of this plant was richer in compounds (42 identified compounds) than the intact one (33 compounds), with a great difference in the percentages of the compounds.

The results obtained by Meffo Dongmo et al. [26] about the composition of *A. citratum* corroborate with what we found to the extent that the major compound present in both studies was geraniol (96.8%).

The differences in chemical composition and percentage of compounds of an EO from a different location can be justified by the fact that many factors such as genetics as well as environmental ones (maturity of fruits, geographical conditions, and climate and seasonal changes) can influence the yield and quantitative–qualitative composition of EOs [40,41].

### 3.2. Insecticidal Activity

Botanical-based pesticides represent a concrete and eco-friendly opportunity for small farmers worldwide, being able to exert their action through multiple mechanisms, therefore limiting resistance development. In this scenario, Africa can play a key role in botanical pesticide discovery, development, and commercialization [42,43]. After a careful analysis of the literature, it has been noted that our knowledge on the insecticidal activity of the *A. citratum*, *M. myristica*, and *X. ethiopica* EOs is patchy. Indeed, the available studies on EOs, only tested the *X. ethiopica* EO against several stored product insect pests [27,28,29,30,31,32,44]. Only a study has been done on *M. myristica* EO, still on stored product beetles [45], while the insecticidal activity of *A. citratum* EO has never been investigated. Further research has been conducted on extracts from the three plants. For example, *M. myristica* ethanolic extracts have been tested against dermestid beetles attacking stored fish [46], while *M. myristica* and *A. citratum* extracts exerted toxic effects on bruchid beetle species developing on stored pulses [44], and acted as antifeedants against *S. littoralis* larvae [47].

Our results pointed out that *M. myristica* and *X. aethiopica* EOs were effective against *M. domestica* adults (♀ LD_50_ 29.1; ♂ LD_50_ 22.1 µg adult^−1^). Bioactivity rates of both EOs are significant, even if still lower if compared to other promising plant EOs, such as the *Carlina acaulis* L. one, recently tested against houseflies [48]. On the other hand, EOs from these plants are much more available [49,50] and at the same time are characterized by a high extraction yield, which ranges between 4–5%.

The insecticidal effectiveness of EOs depends on many factors, such as the content of major constituents and their synergistic or antagonistic relationships, post-application conditions, and/or the modes of action of the major compounds [51,52]. Also in our case, it was found that the effectiveness of EOs was in line with the effectiveness of most substances.

Against houseflies, the EO from *M. myristica* and its major compound *p*-cymene showed comparable effectiveness, while other major compounds performed better in terms of LD_50_ while LD_90_ values were close to those calculated for EOs. In addition, on *S. littoralis* larvae, the *M. myristica* EO was the most effective one, with the major constituent geraniol, showing higher toxicity if compared to the other tested molecules. Last, the EOs from *M. myristica* and *X. aethiopica*, as well as the major constituents *p*-cymene and α-phellandrene, were the most toxic against *C. quinquefasciatus* larvae. Although the relatively good insecticidal effectiveness of the EOs tested by us on the three insect species was found, it will be important to study other options to make the effectiveness of EOs more efficient, so that the lowest possible amount of active substance is applied in practice. An increase in biological effectiveness can be achieved, for example, by using suitable formulation methods, which include encapsulation or nanoemulsion [8,53,54]. These methods can both extend the persistence time and significantly increase the insecticidal efficacy of EOs themselves. However, it will be equally important to study the effect of sublethal doses or concentrations on target and non-target organisms [55]. It was found that even a sublethal concentration or a short period of exposure to EOs can subsequently significantly reduce the vitality of insects [53,56,57]. Thanks to this phenomenon, not only the high mortality of treated larvae or adults can occur, but also the fertility of the next generation can be significantly reduced, and this can lead to a significant reduction in the number of pests or vectors.

Although EOs are generally considered environmentally safe [6,58,59,60,61], our further research will be directed to testing the effect of EOs on selected characteristics of non-target organisms, with the aim of confirming the environmental safety of botanical insecticide applications based on our selected EOs. It will also be important to study the possibility of increasing the content of EOs in plants, on the one hand by using suitable elicitation methods and on the other hand by developing more profitable cultivation technologies, and technique of extractions [62,63,64] that will lead to a higher yield of EOs, like the case of other aromatic plants.

Overall, there is still a long way ahead of us, which will lead from the basic screening we performed to the development and production of botanical insecticides based on EOs from *M. myristica* and *X. aethiopica*. However, this does not change the fact that we have managed to select EOs that may potentially lead to the development and production of effective botanical insecticides for African smallholders [43].

## 4. Materials and Methods

### 4.1. Plant Material and Essential Oil Extraction

The plant material was bought in a local market of Yaounde (3°52′00″ N, 11°31′00″ E, 726 m a.s.l.), Cameroon, in December 2019. The seeds from the three aromatic plants were dried away from the sun at room temperature. Plant specimens were identified by one of us (N. Tsabang). The dried seeds (500 g for *M. myristica*, 584 g for *X. aethiopica*, and 550 g for *A. citratum*) were ground to reduce them into smaller pieces, then inserted in a 10 L Pyrex flask, which was filled with 6 L of distilled water. EOs were obtained by hydrodistillation using a Clevenger-type apparatus for 4 h. The calculation of the oil yields was based on a dry weight (*w*/*w*) matter.

### 4.2. GC-MS Analysis

The *M. myristica*, *X. aethiopica*, and *A. citratum* EOs were prepared by a 1:100 dilution with hexane and analyzed with an Agilent 6890N–5973N GC–MS system operating in the EI mode at 70 eV, using a HP-5MS (5% phenylmethylpolysiloxane, length 30 m, internal diameter 0.25 mm, film thickness 0.1 µm; J & W Scientific, Folsom, CA, USA) capillary column. The total duration of the run was around 66 min with the following temperature program: 60 °C for 5 min, afterward up to 220 °C at 4 °C min^−1^, then up to 280 °C at 11 °C min^−1^ and maintained for 15 min. The carrier gas used in this analysis was helium at a flow rate of 1 mL min^−1^. The injection volume was 2 μL and the split ratio 1:50. The range of acquisition was 29–400 *m z*^−1^. The combination of linear retention indices (RIs) and mass spectra (MS) with those appearing in libraries such as Adams (2007), FFNSC2 (2012), and NIST17 (2017) was the method used for the peak identification unless no analytical standard (purchased from Merck, Milan, Italy) was available. The analytical standards of the major EO components, namely geraniol, sabinene, α-pinene, *p*-cymene, α-phellandrene, and β-pinene, were purchased from Merck (Milan, Italy). Relative peak area percentage for each identified compound was extracted from the total area in the chromatogram without using correction factors.

### 4.3. Insects

Insects used for the tests were obtained from established laboratory colonies, reared under controlled conditions for >20 generations. Uniform larvae of *S. littoralis* (third instar, mean larval weight 12 ± 3 mg), *C. quinquefasciatus* larvae (third instar), and adults of *M. domestica* (males and females, 3–5 days old) were selected for the experiments. The rearing methods of the three species mentioned above were recently described by Benelli et al. [65]). All the species were maintained at 25 ± 1 °C, 70 ± 3% R.H., and 16:8 h (L:D). All below described experiments were carried out under the same conditions.

### 4.4. Insecticidal Activity

Contact toxicity of *M. myristica*, *X. aethiopica*, and *A. citratum* EOs was evaluated by applying them, as well as their major constituents (i.e., geraniol, sabinene, α-pinene, *p*-cymene, α-phellandrene, and β-pinene), topically on the pronotum of *M. domestica* males and females, as well as on *S. littoralis* larvae. The EOs or major compounds were dissolved in acetone to obtain a concentration series. Subsequently, 1 µL was applied on each insect using a micro-electric applicator (HandyStep Electronic, Brand, Turnov, Czech Republic) to treat the CO_2_-anesthetized *M. domestica* or *S. littoralis*. The tested doses were 20, 30, 50, 80, 100, 120, 140, 160, 180, 200, and 250 µg adult^−1^ or 20, 40, 60, 80, 100, 120, 150, 180, 210, and 250 µg larva^−1^, respectively. Certified acetone (Sigma-Aldrich, Darmstadt, Germany) was used as the negative control treatment in the experiments. After the application, moth larvae and housefly adults were moved to rearing containers sized 15 cm × 12 cm × 8 cm with perforated lids (at 25 ± 1 °C, 70 ± 3% R.H. and 16:8 h (L:D)), containing their usual food.

In the assays focusing on *C. quinquefasciatus* larvae, *M. myristica*, *X. aethiopica*, and *A. citratum* EOs and their major constituents (i.e., geraniol, sabinene, α-pinene, *p*-cymene, α-phellandrene, and β-pinene) were diluted in dimethyl sulfoxide (DMSO) and then tested following WHO (1996) with minor modifications by Pavela and Sedlak [52] and Pavela et al. [48]; the tested concentrations were 20, 30, 40, 50, 70, 80, 100, 120, and 140 µg mL^−1^. Distilled water with the same amount of DMSO as that used for dissolving the EOs was used as negative control.

To calculate the lethal doses/concentrations for *M. myristica*, *X. aethiopica*, and *A. citratum* EOs and their main compounds on each insect target, we used a minimal series of at least five different doses/concentrations that resulted in mortality rates in the range of 10–90%. The experiment was replicated four times in total (20 insects per replication). Insect mortality was assessed 24 h after treatment. In insecticidal experiments, mortality was corrected, where needed, through the Abbott’s formula [66], LC_50_ and LC_90_ and the associated 95% confidence limits were estimated by probit analysis [67].

## 5. Conclusions

In this work, three EOs extracted from the Cameroonian plants *M. myristica*, *X. aethiopica*, and *A. citratum* were analyzed, and their insecticidal activity was evaluated. Their efficacy against *M. domestica*, *S. littoralis*, and *C. quinquefasciatus* proved to be strongly influenced by the synergistic and antagonistic interactions of the major constituents. In particular, *M. myristica* and *X. aethiopica* and their major components *p*-cymene and α-phellandrene resulted the most active against both houseflies and *C. quinquefasciatus*, while *S. littoralis* larvae showed sensitivity to *M. myristica* EO. Due to their activity and wide distribution in the African continent, these EOs have a great potential to develop insecticide products on a large scale. Future steps in this direction will be the encapsulation of the EOs in nanoformulations and the evaluation of the effect of sublethal doses or concentrations on target and non-target organisms.

## Figures and Tables

**Table 1:** Chemical composition of the essential oils extracted from seeds of *Monodora myristica*, *Aframomum citratum*, and *Xylopia aethiopica*.

			Relative % in Essential Oil			
Component ^a^	RI Calc. ^b^	RI Lit. ^c^	*M. myristica* ^d^	*X. aethiopica*	*A. citratum*	Method of Identification ^e^
α-Thujene	920	924	3.1 ± 0.6	1.9 ± 0.4	tr ^f^	RI,MS
α-Pinene	925	932	7.6 ± 1.2	9.6 ± 1.6	1.0 ± 0.2	Std
Camphene	938	946		0.1 ± 0.0		Std
Thuja-2,4(10)-diene	944	953		tr		RI,MS
Sabinene	966	969	0.1 ± 0.0	26.1 ± 3.1		Std
β-Pinene	968	974	0.3 ± 0.0	17.4 ± 1.9	5.4 ± 0.9	Std
Myrcene	988	988	4.3 ± 0.8	0.2 ± 0.0	0.1 ± 0.0	Std
δ-2-carene	998	1001	0.9 ± 0.0		0.1 ± 0.0	RI,MS
α-Phellandrene	1002	1002	32.3 ± 3.6	0.3 ± 0.0		Std
α-Terpinene	1013	1014	0.1 ± 0.0	1.9 ± 0.4		Std
*p*-Cymene	1021	1020	32.8 ± 3.0	1.1 ± 0.2	0.1 ± 0.0	Std
Limonene	1024	1024	4.4 ± 0.9		0.3 ± 0.1	Std
β-Phellandrene	1024	1025		6.2 ± 1.2		Std
1,8-Cineole	1026	1026		3.6 ± 0.7	0.7 ± 0.2	Std
(*Z*)-β-Ocimene	1036	1032	0.3 ± 0.0	1.2 ± 0.3	tr	Std
(*E*)-β-Ocimene	1046	1044	0.1 ± 0.0	0.1 ± 0.0	0.3 ± 0.0	Std
α-Terpinene	1054	1054	0.1 ± 0.0	3.2 ± 0.6	tr	Std
*cis*-Sabinene hydrate	1062	1065		1.3 ± 0.3		RI,MS
*cis*-Linalool oxide	1069	1067			tr	RI,MS
Terpinolene	1084	1086	tr	0.6 ± 0.2		Std
*trans*-Sabinene hydrate	1093	1098		0.8 ± 0.2		RI,MS
Linalool	1100	1095	1.9 ± 0.4	0.1 ± 0.0	2.4 ± 0.5	Std
*cis*-*p*-Menth-2-en-1-ol	1117	1118	0.2 ± 0.0	0.3 ± 0.0		RI,MS
α-Campholenal	1122	1122	tr	0.1 ± 0.0		RI,MS
*allo*-Ocimene	1128	1128		0.1 ± 0.0		RI,MS
*trans*-Pinocarveol	1131	1135		0.4 ± 0.1	tr	Std
*trans*-*p*-Menth-2-en-1-ol	1135	1136	0.1 ± 0.0	0.2 ± 0.0		RI,MS
*trans*-Verbenol	1140	1140		0.1 ± 0.0		RI,MS
Pinocarvone	1156	1160		0.2 ± 0.0		RI,MS
Borneol	1159	1165	0.1 ± 0.0	tr		Std
*p*-Mentha-1,5-dien-8-ol	1164	1166		tr		RI,MS
*cis*-Pinocamphone	1167	1172		tr		RI,MS
Terpinen-4-ol	1172	1174		6.1 ± 1.1	tr	Std
*p*-Cymen-8-ol	1183	1179	0.1 ± 0.0			RI,MS
Cryptone	1181	1183		0.1 ± 0.0		RI,MS
α-Terpineol	1186	1186	0.5 ± 0.1	1.0 ± 0.2	0.3 ± 0.0	RI,MS
Myrtenal	1189	1195		0.2 ± 0.0	tr	Std
Myrtenol	1191	1194		0.4 ± 0.1		Std
*trans*-Piperitol	1203	1207	0.1 ± 0.0			RI,MS
Verbenone	1204	1204		0.1 ± 0.0		RI,MS
Cuminaldehyde	1236	1238		tr		RI,MS
Neral	1239	1235			0.5 ± 0.2	RI,MS
Carvotanacetone	1243	1244	0.1 ± 0.0			RI,MS
Geraniol	1264	1249			85.6 ± 2.8	Std
Geranial	1272	1264			1.9 ± 0.4	Std
Carvacrol	1303	1298	0.6 ± 0.2			Std
δ-Elemene	1331	1335		1.8 ± 0.4		RI,MS
α-Cubebene	1343	1345		0.1 ± 0.0		RI,MS
α-Ylangene	1362	1373		0.2 ± 0.0		RI,MS
α-Copaene	1367	1374	0.1 ± 0.0	0.4 ± 0.1		RI,MS
β-Cubebene	1382	1387		0.1 ± 0.0		RI,MS
β-Elemene	1385	1389		0.2 ± 0.0		Std
Geranyl acetate	1385	1379			0.8 ± 0.2	RI,MS
Cyperene	1387	1398			0.1 ± 0.0	RI,MS
(*E*)-Caryophyllene	1408	1417			tr	Std
*cis*-α-Bergamotene	1408	1411	0.1 ± 0.0			RI,MS
β-Ylangene	1408	1419		0.2 ± 0.0		RI,MS
α-Santalene	1412	1416	0.5 ± 0.1			RI,MS
β-Copaene	1419	1430		0.1 ± 0.0		RI,MS
γ-Elemene	1426	1434		0.3 ± 0.0		RI,MS
*trans*-α-Bergamotene	1430	1432	tr			RI,MS
6,9-Guaiadiene	1434	1442		0.1 ± 0.0		RI,MS
α-Humulene	1442	1452	tr	0.1 ± 0.0		Std
Germacrene D	1471	1484		9.7 ± 1.6		RI,MS
Bicyclogermacrene	1486	1500		0.5 ± 0.2		RI,MS
α-Muurolene	1492	1500	0.1 ± 0.0	tr		RI,MS
δ-Amorphene	1498	1511		0.1 ± 0.0		RI,MS
γ-Cadinene	1504	1513	0.3 ± 0.0	0.1 ± 0.0		RI,MS
δ-Cadinene	1516	1522	1.2 ± 0.3	0.3 ± 0.1		RI,MS
α-Cadinene	1528	1537	tr			RI,MS
Germacrene B	1544	1559		0.3 ± 0.1		RI,MS
*epi*-α-Cadinol	1631	1638	0.1 ± 0.0			RI,MS
*epi*-α-Muurolol	1645	1640	0.1 ± 0.0			RI,MS
Manool oxide	1991	1987		0.1 ± 0.0		RI,MS
Total identified (%)			92.9	99.6	99.9	
Number of identified compounds			35	55	23	
Grouped compounds (%)						
Monoterpene hydrocarbons			86.5	69.8	7.4	
Oxygenated monoterpenes			3.8	15.0	91.5	
Sesquiterpene hydrocarbons			2.4	14.6	0.2	
Oxygenated sesquiterpenes			0.2	-	-	
Others			tr	0.2	0.9	

^a^ Order of components according to their elution from a HP-5MS column. ^b^ RI calc.: calculated linear retention index using a mix of *n*-alkanes (C8–C30). ^c^ RI lit.: retention index reported from the literature. ^d^ Peak area percentage as the mean of three injections (different solution preparations) ± standard deviation. ^e^ Method of identification: Std, comparison of RI and MS with that of analytical standard; RI, coherence of the linear retention index with those reported in literature; MS, mass fragmentation overlapping with those stored in commercial libraries. ^f^ tr: trace.

**Table 2:** Insecticidal activity of *Monodora myristica*, *Aframomum citratum*, and *Xylopia aethiopica* seed essential oils and their major constituents on *Musca domestica* adults.

	*Musca domestica*—Female							*Musca domestica*—Male						
	LD_50_ (µg adult^−1^)	CI_95_	LC_90_ (µg adult^−1^)	CI_95_	*χ* ^2^	*df*	*p*-value	LD_50_ (µg adult^−1^)	CI_95_	LC_90_ (µg adult^−1^)	CI_95_	*χ* ^2^	*df*	*p*-Value
Essential oils														
*Monodora myristica*	29.1	18.5–42.8	137.6	128.5–152.7	4.763	4	0.321	22.1	15.7–26.9	127.6	111.2–136.7	2.465	4	0.144
*Aframomum citratum*	48.9	43.5–65.7	373.5	301.8–421.7	2,574	5	0.253	80.5	69.7–91.5	160.2	155.7–189.5	2.215	4	0.286
*Xylopia aethiopica*	30.7	22.5–40.8	164.2	135.7–178.9	1.682	3	0.641	61.5	55.7–65.7	178.5	156.7–192.9	5.125	4	0.562
Major compounds														
Geraniol	151.5	111.5–182.7	288.7	232.5–302.8	3.455	3	0.452	23.5	17.6–29.3	126.1	111.5–148.9	1.518	3	0.687
Sabinene	109.7	85.7–122.5	213.8	195.7–252.7	3.452	3	0.652	10.4	8.2–15.9	117.5	98.6–135.9	2.452	3	0.128
α-Pinene	69.7	51.5–78.8	254.7	212.2–278.9	2.751	3	0.428	8.6	7.2–15.9	100.2	87.5–120.9	4.256	3	0.318
*p*-Cymene	28.4	16.5–31.9	131.7	118.5–145.7	5.123	4	0.251	32.6	28.9–51.6	145.6	126.9–158.7	2.246	3	0.257
α-Phellandrene	43.5	35.5–48.9	187.9	175.7–201.5	3.456	4	0.425	46.7	39.7–56.2	178.9	156.8–193.3	1.152	3	0.562
β-Pinene	56.1	42.8–65.3	316.5	289.7–324.7	2.245	4	0.156	39.7	33.5–42.8	189.7	165.9–195.5	2.852	4	0.349

**Table 3:** Insecticidal activity of *Monodora myristica*, *Aframomum citratum*, and *Xylopia aethiopica* essential oils and their major constituents on *Spodoptera littoralis* third instar larvae.

	LC_50_ (µg larva^−1^)	CI_95_	LC_90_ (µg larva^−1^)	CI_95_	*χ* ^2^	*df*	*p*-Value
Essential oils							
*Monodora myristica*	29.3	21.5–37.1	123.5	102.5–148.7	1.663	3	0.645
*Aframomum citratum*	31.1	23.6–39.7	130.2	118.7–142.6	1.076	3	0.782
*Xylopia aethiopica*	60.3	52.8–71.5	155.6	128.7–165.5	1.721	3	0.632
Major compounds							
Geraniol	25.2	19.7–28.6	83.2	72.9–105.6	1.478	3	0.687
Sabinene	45.1	38.9–52.3	240.4	201.8–259.7	1.378	3	0.711
α-Pinene	123.5	111.5–138.7	198.7	179.6–222.1	2.528	3	0.477
*p*-Cymene	52.3	44.1–60.5	112.2	98.7–125.9	2.147	3	0.542
α-Phellandrene	54.6	43.7–66.1	143.7	132.6–169.5	1.642	3	0.649
β-Pinene	84.5	72.8–101.8	226.5	212.5–257.8	3.458	3	0.117

**Table 4:** Insecticidal activity of *Monodora myristica*, *Aframomum citratum*, and *Xylopia aethiopica* essential oils and their major constituents on *Culex quinquefasciatus* third instar larvae.

	LC_50_ (µg mL^−1^)	CI_95_	LC_90_ (µg mL^−1^)	CI_95_	*χ* ^2^	*df*	*p*-Value
Essential oils							
*Monodora myristica*	35.3	25.1–42.3	66.1	55.8–72,4	3.236	3	0.356
*Aframomum citratum*	82.7	75.1–90.3	160.1	149.8–196.5	3.003	3	0.391
*Xylopia aethiopica*	47.0	38.7–52.2	78.4	63.9–92.7	5.245	3	0.079
Major compounds							
Geraniol	98.1	91.5–104.8	153.3	138.9–165.7	1.011	3	0.798
Sabinene	64.4	60.1–68.5	102.3	93.5–116.7	0.311	3	0.998
α-Pinene	74.5	66.1–81.5	144.7	128.5–172.7	1.183	3	0.686
*p*-Cymene	26.8	21.5–37.9	56.5	42.7–75.9	3.526	3	0.161
α-Phellandrene	36.8	25.7–51.3	85.1	72.8–95.9	3.512	3	0.318
β-Pinene	66.1	61.3–70.5	109.3	100.2–123.1	0.089	4	0.999

## Data Availability

The data presented in this study are available on request from the corresponding authors.
